# Supplementary material for: An optimised multi-arm multi-stage clinical trial design for unknown variance
Source: Contemp Clin Trials. 2018 Apr;67:116–20. doi: 10.1016/j.cct.2018.02.011 (PMC5886309; doi:10.1016/j.cct.2018.02.011)
Supplement: Supplementary file 1 — Supplementary material [file mmc1.docx]

**Supplementary Material for “An optimised multi-arm multi-stage clinical trial design for unknown variance”**

1. **The sets** $\boldsymbol{\Xi}_{\mathbf{sim}}$**,** $\boldsymbol{\Xi}_{\mathbf{sep}}$**,** $\boldsymbol{\Xi}_{\mathbf{rej}}$**, and** $\boldsymbol{\Xi}_{\mathbf{1}}$

In Section 2 of the paper, we defined the vectors $\boldsymbol{\psi}=\left( \psi_{1},\ldots,\psi_{K} \right)$ and $\boldsymbol{\omega=}\left( \omega_{1},\ldots,\omega_{K} \right)$, as

- $\psi_{k}\in\left\{ 0,1 \right\}$, with $\psi_{k}=1$ if $H_{0}^{\left( k \right)}$ is rejected, and $\psi_{k}=0$ otherwise;
- $\omega_{k}\in\left\{ 1,\ldots,J \right\}$, with $\omega_{k}=j$ if $j$ is the analysis at which $H_{0}^{\left( k \right)}$ is rejected, accepted, or the whole trial is stopped and no decision on $H_{0}^{\left( k \right)}$ is made.

We used these vectors in defining the following four sets

$$\Xi_{\mathrm{sim}}=\left\{ \boldsymbol{(\omega,\psi)}\in\left\{ 1,\ldots,J \right\}^{K}\times\left\{ 0,1 \right\}^{K} : \mathrm{if} \sum_{k=1}^{K} \mathbb{I}\left\{ \omega_{k}\leq j \right\}\mathbb{I}\left\{ \psi_{k}=1 \right\}\geq1 \mathrm{then} \sum_{k=1}^{K} \mathbb{I}\left\{ \omega_{k}>j \right\}=0 \forall j \right\},$$

$$\Xi_{\mathrm{sep}}=\left\{ \boldsymbol{(\omega,\psi)}\in\left\{ 1,\ldots,J \right\}^{K}\times\left\{ 0,1 \right\}^{K} \right\},$$

$$\Xi_{\mathrm{rej}}=\left\{ \boldsymbol{(\omega,\psi)}\in\Xi: \sum_{k=1}^{K} \mathbb{I}\left\{ \psi_{k}=1 \right\}>0 \right\},$$

$$\Xi_{1}=\left\{ \boldsymbol{(\omega,\psi)}\in\Xi\mathbb{: I}\left\{ \psi_{1}=1 \right\}=1 \right\}.$$

As discussed, $\Xi_{\mathrm{sim}}$ and $\Xi_{\mathrm{sep}}$ represent the set of possible $\boldsymbol{\omega}\boldsymbol{,\psi}$ combinations when using the simultaneous and separate stopping rules respectively. Whilst, for $\Xi\in\{\Xi_{\mathrm{sim}},\Xi_{\mathrm{sep}}\}$set according to the chosen stopping rule, $\Xi_{\mathrm{rej}}$ and $\Xi_{1}$ are respectively the subsets of the $\Xi$ such that at least one null hypothesis, or $H_{0}^{\left( 1 \right)}$, is rejected. Here, to try to elucidate what these sets are more clearly we consider a simple example.

Suppose that we conduct a three-arm ($K=2$) two-stage trial ($J=2$). Then, we must have that $\psi_{1}$, $\psi_{2}\in\left\{ 0,1 \right\}$ and $\omega_{1},\omega_{2}\in\left\{ 1,2 \right\}$. If we use a separate stopping rule then any combination of these values is possible, and so we have

$$\Xi_{\mathrm{sep}}\boldsymbol{=\{}\left( 1,1,0,0 \right),\left( 1,1,0,1 \right),\left( 1,1,1,0 \right),\left( 1,1,1,1 \right),$$

$$\left( 1,2,0,0 \right),\left( 1,2,0,1 \right),\left( 1,2,1,0 \right),\left( 1,2,1,1 \right),$$

$$\left( 2,1,0,0 \right),\left( 2,1,0,1 \right),\left( 2,1,1,0 \right),\left( 2,1,1,1 \right),$$

$$\left( 2,2,0,0 \right),\left( 2,2,0,1 \right),\left( 2,2,1,0 \right),\left( 2,2,1,1 \right)\boldsymbol{\}.}$$

For the simultaneous stopping rule we only proceed to stage 2 if a null hypothesis is not rejected at the end of stage 1. Thus, we have

$$\Xi_{\mathrm{sim}}\boldsymbol{=\{}\left( 1,1,0,0 \right),\left( 1,1,0,1 \right),\left( 1,1,1,0 \right),\left( 1,1,1,1 \right),$$

$$\left( 1,2,0,0 \right),\left( 1,2,0,1 \right),\left( 2,1,0,0 \right),\left( 2,1,1,0 \right),$$

$$\left( 2,2,0,0 \right),\left( 2,2,0,1 \right),\left( 2,2,1,0 \right),\left( 2,2,1,1 \right)\boldsymbol{\}}$$

The condition in the general definition of $\Xi_{\mathrm{sim}}$ above is constructed to remove those scenarios possible when using a separate stopping rule, which become impossible for the simultaneous stopping rule.

Finally, $\Xi_{\mathrm{rej}}$ and $\Xi_{1}$ are then easy to construct from the above by extracting those scenarios where at least one of $\psi_{1}$ and $\psi_{2}$ are equal to 1, or $\psi_{1}=1$, respectively. For example, for the simultaneous stopping rule

$$\Xi_{\mathrm{rej}}\boldsymbol{=\{}\left( 1,1,0,1 \right),\left( 1,1,1,0 \right),\left( 1,1,1,1 \right),\left( 1,2,0,1 \right),$$

$$\left( 2,1,1,0 \right),\left( 2,2,0,1 \right),\left( 2,2,1,0 \right),\left( 2,2,1,1 \right)\},$$

$$\Xi_{1}=\left\{ \left( 1,1,1,0 \right),\left( 1,1,1,1 \right),\left( 2,1,1,0 \right),\left( 2,2,1,0 \right),\left( 2,2,1,1 \right) \right\}\boldsymbol{.}$$

The conditions given in the general definitions of $\Xi_{\mathrm{rej}}$ and $\Xi_{1}$ again ensure that we restrict to only the particular scenarios of interest.

1. **Design assuming known variance**

Here, we summarise how we can evaluate the operating characteristics of a design in the case that the variance parameter $\sigma$ is known. In this instance, the $T_{kj}=T_{kj}\left( \sigma\right)$ are together multivariate normal. Specifically

$$\mathbb{E}\left( T_{kj} \right)=\theta_{k}\sqrt{\frac{jn}{2\sigma^{2}}},$$

$$\mathrm{Cov}\left( T_{kj_{1}},T_{kj_{2}} \right)=\sqrt{\frac{j_{1}}{j_{2}}}, j_{1}\leq j_{2},$$

$$\mathrm{Cov}\left( T_{k_{1}j_{1}},T_{k_{2}j_{2}} \right)=\frac{1}{2}\sqrt{\frac{j_{1}}{j_{2}}}, j_{1}\leq j_{2}, k_{1}\neq k_{2}.$$

We can evaluate the FWER and power for any particular choices of $n$, $\boldsymbol{e}$ and $\boldsymbol{f}$ using the formulae

$$\alpha_{\mathrm{FWER}}=\sum_{\left( \boldsymbol{\omega,\psi} \right)\in\Xi_{\mathrm{rej}}} \mathbb{P}\left( \boldsymbol{\omega,\psi} | \boldsymbol{0} \right)$$

$$1-\beta_{\mathrm{power}}=\sum_{\left( \boldsymbol{\omega,\psi} \right)\in\Xi_{1}} \mathbb{P}\left( \boldsymbol{\omega,\psi} | \boldsymbol{\delta} \right),$$

$$\mathrm{ESS}\left( \boldsymbol{\theta} \right)=\sum_{\left( \boldsymbol{\omega,\psi} \right)\in\Xi} n\left( \max_{k} \omega_{k}+\sum_{k=1}^{K} \omega_{k} \right)\mathbb{P}\left( \boldsymbol{\omega,\psi} | \boldsymbol{\theta} \right),$$

from the main part of the paper. The values of $\mathbb{P}\left( \boldsymbol{\omega}\boldsymbol{,\psi} | \boldsymbol{\theta} \right)$ are evaluated using multivariate normal integration. The range of integration for the variable corresponding to the test statistic $T_{kj}$ is set using $\boldsymbol{\omega}$ and $\boldsymbol{\psi}$, and depends on the chosen stopping rule. Precisely, the lower range of the integral, $l_{kj}$, and the upper range, $u_{kj}$, are

$$l_{kj}=\left\{ \begin{aligned} e_{j} : \psi_{k}=1,\omega_{k}=j, \\ -\infty:\left\{ \omega_{k}<j \right\}\cup\left\{ \psi_{k}=0,\omega_{k}=j \right\} \\ f_{j} : otherwise, \end{aligned} \right.,$$

$$u_{kj}=\left\{ \begin{aligned} e_{j} : \left\{ \omega_{k}>j \right\}\cup\left\{ \psi_{k}=0,\omega_{k}=j,\max_{m\in\left\{ 1,\ldots,K \right\}} \omega_{m}=j,\sum_{m=1}^{K} \mathbb{I}\left\{ \psi_{m}=1 \right\}\geq S \right\}, \\ \infty: \left\{ \omega_{k}<j \right\}\cup\left\{ \psi_{k}=1,\omega_{k}=j \right\}, \\ f_{j} : otherwise, \end{aligned} \right.$$

where $S=1$ for the simultaneous stopping rule and $S=K$ for the separate stopping rule.

Finally, denoting the mean of $\left( T_{11},\ldots,T_{K1},T_{12},\ldots,T_{K2},\ldots,T_{1J},\ldots,T_{KJ} \right)^{\top}$ by $\boldsymbol{\mu}$ and its covariance by $\Sigma$ (which can both be computed using the formulae above) we have

$$\mathbb{P}\left( \boldsymbol{\omega,\psi} | \boldsymbol{\theta} \right)=\int_{l_{11}}^{u_{11}} \ldots\int_{l_{K1}}^{u_{K1}} \int_{l_{12}}^{u_{12}} \ldots\int_{l_{K2}}^{u_{K2}} \ldots\int_{l_{1J}}^{u_{1J}} \ldots\int_{l_{KJ}}^{u_{KJ}} \phi\left\{ \boldsymbol{x},\boldsymbol{\mu},\Sigma\right\}dx_{11}\ldots dx_{KJ},$$

where $\phi\left\{ \boldsymbol{x},\boldsymbol{\mu},\Sigma\right\}$ is the probability density function of a multivariate normal distribution with mean $\boldsymbol{\mu}$ and covariance $\Sigma$, evaluated at $\boldsymbol{x=}\left( x_{11},\ldots,x_{KJ} \right)$.

1. **Pairwise Error-rate**

In the main part of the paper we presented the results of a simulation study evaluating the performance of four approaches in two scenarios, for two considered stopping rules. Here, we discuss corresponding results for the PWER, which we estimate using the following formula

$$\hat{\alpha}_{\mathrm{PWER}}=\frac{1}{R}\sum_{r=1}^{R} \mathbb{I}\left\{ \left( \boldsymbol{\omega}_{r},\boldsymbol{\psi}_{r} \right)\in\Xi_{1} \right\},$$

with data simulated under the scenario that $\boldsymbol{\theta=0}$. Our results are presented in Supplementary Table 1.

As would be expected, the PWER is lower for the simultaneous stopping rule designs than the analogous value for the separate stopping rule designs. This is a consequence of the fact that the former allows for the possibility that we incorrectly reject $H_{0}^{\left( 1 \right)}$ after the second stage, even when $H_{0}^{\left( 2 \right)}$ has been rejected after the first stage.

Following our results for the FWER, it is clear that Approaches 3 and 4 substantially reduce the value of the PWER when $\sigma_{T}^{2}>\sigma^{2}$.

| Approach | Scenario 1 | | | | |  | Scenario 2 | | | | | |
| --- | --- | --- | --- | --- | --- | --- | --- | --- | --- | --- | --- | --- |
|  | $\sigma_{T}^{2}$ | | | | |  | $\sigma_{T}^{2}$ | | | | | |
|  | $\boldsymbol{0.25}$ | $\boldsymbol{0.5}$ | $\boldsymbol{1.0}$ | $\boldsymbol{2.0}$ | $\boldsymbol{4.0}$ |  | $\boldsymbol{0.25}$ | $\boldsymbol{0.5}$ | $\boldsymbol{1.0}$ | $\boldsymbol{2.0}$ | $\boldsymbol{4.0}$ | |
| Simultaneous stopping rule designs | | | | | | | | | | | | |
| A1 | 0.0000 | 0.0012 | 0.0181 | 0.0733 | 0.1538 |  | 0.0000 | 0.0014 | 0.0186 | 0.0745 | 0.1567 | |
| A2 | 0.0191 | 0.0193 | 0.0190 | 0.0193 | 0.0196 |  | 0.0221 | 0.0213 | 0.0213 | 0.0217 | 0.0212 | |
| A3 | 0.0183 | 0.0186 | 0.0183 | 0.0186 | 0.0188 |  | 0.0197 | 0.0186 | 0.0190 | 0.0191 | 0.0188 | |
| A4 | 0.0191 | 0.0184 | 0.0188 | 0.0187 | 0.0183 |  | 0.0197 | 0.0190 | 0.0190 | 0.0191 | 0.0188 | |
| Separate stopping rule designs | | | | | | | | | | | |  |
| A1 | 0.0000 | 0.0013 | 0.0189 | 0.0805 | 0.1691 |  | 0.0000 | 0.0013 | 0.0196 | 0.0802 | 0.1723 | |
| A2 | 0.0200 | 0.0210 | 0.0200 | 0.0202 | 0.0208 |  | 0.0222 | 0.0226 | 0.0226 | 0.0232 | 0.0229 | |
| A3 | 0.0193 | 0.0201 | 0.0194 | 0.0194 | 0.0200 |  | 0.0194 | 0.0200 | 0.0197 | 0.0202 | 0.0199 | |
| A4 | 0.0195 | 0.0202 | 0.0188 | 0.0197 | 0.0204 |  | 0.0204 | 0.0201 | 0.0196 | 0.0200 | 0.0203 | |

**Supplementary Table 1:** The estimated pairwise error-rate ($\hat{\alpha}_{\mathrm{PWER}}$) when $\boldsymbol{\theta}\boldsymbol{=0}$ of the four considered approaches (A1-A4) are shown as the true variance $\sigma_{T}^{2}$ varies, for the two considered trial design scenarios, and the two considered stopping rules. The rejection rate values are given to four decimal places.
